# Changes in academic performance in the online, integrated system-based curriculum implemented due to the COVID-19 pandemic in a medical school in Korea

**DOI:** 10.3352/jeehp.2021.18.24

**Published:** 2021-09-23

**Authors:** Do-Hwan Kim, Hyo Jeong Lee, Yanyan Lin, Ye Ji Kang

**Affiliations:** Department of Medical Education, Hanyang University College of Medicine, Seoul, Korea; Hallym University, Korea

**Keywords:** Academic performance, COVID-19, Distance education, Undergraduate medical education, Republic of Korea

## Abstract

**Purpose:**

This study examined how students’ academic performance changed after undergoing a transition to online learning during the coronavirus disease 2019 (COVID-19) pandemic, based on the test results of 16 integrated courses conducted in 3 semesters at Hanyang University College of Medicine in Korea.

**Methods:**

For the 16 required courses that formed an integrated system-based curriculum running for 3 semesters, the major examinations’ raw scores were collected for each student. Percent-correct scores were used in the subsequent analysis. We used the t-test to compare grades between 2019 and 2020, and the Cohen D was calculated as a measure of effect size. The correlation of scores between courses was calculated using Pearson correlation coefficients.

**Results:**

There was a significant decrease in scores in 2020 for 10 courses (62.5%). While most of the integrated system-based curriculum test scores showed strong correlations, with coefficients of 0.6 or higher in both 2019 and 2020, the correlation coefficients were generally higher in 2020. When students were divided into low, middle, and high achievement groups, low-achieving students consistently showed declining test scores in all 3 semesters.

**Conclusion:**

Our findings suggest that the transition to online classes due to COVID-19 has led to an overall decline in academic performance. This overall decline, which may occur when the curriculum is centered on recorded lectures, needs to be addressed. Further, medical schools need to consider establishing a support system for the academic development of low-achieving students.

## Introduction

### Background/rationale

Medical educators have developed and implemented various teaching methods with the goal of more effectively and efficiently cultivating learners’ abilities as doctors. For example, online learning has been highly anticipated since much of the medical school curriculum consists of didactic lectures that deliver knowledge. However, resistance from participants, ranging from students to professors, as well as barriers regarding institutional resources, policies, and support, slowed the transition from traditional face-to-face classes to online classes [1]. Starting in early 2020, the coronavirus disease 2019 (COVID-19) pandemic led to unexpectedly rapid changes in this domain, as it forced the full-scale implementation of online classes without a face-to-face option.

This transition to online learning can be seen as an educational intervention introduced in the pre-clerkship curriculum, where most of the learning takes place through lecture-based teaching. Program evaluation, therefore, is essential to investigate the impact of the intervention and to improve curricular outcomes. Under these circumstances, outcomes can be assessed using Kirkpatrick’s 4-level evaluation model, which is a framework widely used for program evaluation [2].

In the first level of the model (i.e., reaction), numerous findings have been reported regarding students’ perceptions of online classes and satisfaction with different teaching methods. The results of these studies are quite consistent. For example, according to a recently released meta-analysis, synchronous distance education (SDE) has higher satisfaction ratings than face-to-face classes [3]. Similar results were found in post-COVID-19 surveys, indicating that students prefer online courses over offline courses [4]. Particularly, students prefer classes offering recorded videos over live online lectures, citing “fast viewing” and “pause and resume” as the advantages of recorded videos that cannot be met in either face-to-face or live online lectures [4].

In contrast, in the second level of the model (i.e., learning), there is still insufficient evidence to draw definitive conclusions on how post-COVID-19 online classes differ from conventional ones. Although students recognized that the COVID-19 pandemic “greatly affected” or “considerably affected” their studies [5], in practice, the direction of influence is possibly both positive and negative. The fact that high satisfaction does not necessarily guarantee high academic achievement is another reason why it is difficult to make a convincing argument for academic achievement despite students’ positive responses to online courses [6]. Previous studies have demonstrated that a student’s “feeling of learning” has no association with actual academic achievement, and sometimes they are even negatively correlated [7].

### Objectives

This study aims to examine how students’ academic performance changed after the COVID-19 pandemic based on the test results of 16 integrated courses conducted over 3 semesters at a single medical school in South Korea that underwent the online transition after COVID-19.

## Methods

### Ethics statement

This study was reviewed and approved by the Institutional Review Board of Hanyang University (approval no., HYUIRB-202105-023-1).

### Study design

This was a comparative observational study, involving a comparison of 16 integrated, system-based courses before and after the transition to online classes.

### Setting

This study was conducted at Hanyang University College of Medicine (HYUCM), a private medical school in Seoul, South Korea. The average number of students per year is about 100. HYUCM operates a 6-year undergraduate-entry program, consisting of a 2-year premedical course and a 4-year medical course. The 4-year medical course is divided into three phases. Phase 1 (1 semester) and phase 2 (3 semesters) correspond to the pre-clerkship period, and phase 3 is the clinical clerkship period. In HYUCM, the transition to online teaching was first implemented after COVID-19. Almost all face-to-face classroom lectures were replaced by online recorded videos, while fewer than 5% of classes were conducted as live online lectures.

In HYUCM, phase 2 is an integrated, system-based curriculum that runs for 3 semesters from the second semester of the first year. There are a total of 17 required courses in this period without any student-selected components (Table 1). The course lengths range from 1 to 7 weeks, and they are organized into blocks. To promote the integrated understanding of clinical and anatomical knowledge, most courses include some cadaver dissection sessions. Every course has at least 1 major examination as a summative assessment. Major examinations are conducted up to 3 times depending on the course length, and all examinations consist of multiple-choice questions (MCQ) items or short-answer questions. Each major examination score is added up to constitute about 80%–90% of a course’s final score. Other components such as attendance and evaluation in problem-based learning (PBL) sessions account for the remaining 10%–20%.

### Variables

Students’ performance records in all courses were variables.

### Data sources/measurement

The major examinations’ raw scores were collected for each student. Because the total score was different for each examination, percent-correct scores were used in subsequent analyses. For courses that conducted more than 1 major examination, student achievement was calculated as an average of the percent-correct scores obtained from the examinations. As the aim of this study was to investigate academic achievement in terms of knowledge acquisition, we did not consider other assessment components, such as attendance or PBL scores, which are more intended to assess conscientiousness, communication, or critical thinking rather than knowledge.

### Bias

Given this study included 16 (out of 17) courses throughout 3 semesters of phase 2 and the examination scores from all students, the risk of selection bias was negligible.

### Study size

All medical students’ records in the corresponding courses were included. There was no estimation of sample size.

### Statistical methods

Data were analyzed using IBM SPSS ver. 26.0 (IBM Corp., Armonk, NY, USA). The t-test was used to compare grades between 2019 and 2020, and the Cohen D was calculated as a measure of the effect size. The correlation of scores between courses was calculated using Pearson correlation coefficients. Correlation coefficients were compared after applying the Fisher r-to-z transformation. P-values <0.05 were considered to indicate statistical significance.

## Results

### Difference in test scores between 2019 and 2020

**Fig. 1.**
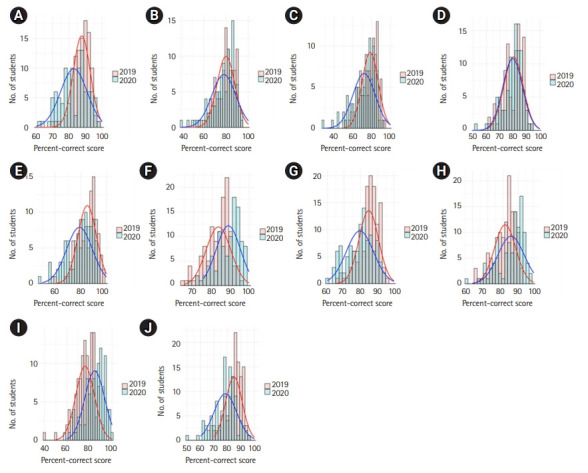
Histogram of examination scores for each course. (A–E) The first year (first semester of phase 2). (F–J) The second year (second semester of phase 2). (A) Introduction to Clinical Medicine, (B) Musculoskeletal System, (C) Medical Neuroscience, (D) Neuropsychiatry and Behavioral Science, (E) Sensory System, (F) Respiratory Medicine, (G) Gastroenterology, (H) Endocrinology and Metabolism, (I) Reproductive Medicine, (J) Kidney and Urinary Tract, (K) Immunology, (L) Infectious Disease, (M) Hematology and Oncology, (N) Occupational Disease and Injury, (O) Birth and Growth, and (P) Geriatrics.

When comparing the test results from the 16 courses, a significant decrease in scores was found in 10 courses (62.5%) in 2020 (Table 2, Dataset 1). There was no significant difference in 3 courses, and a significant increase was found in 3 courses. Using the Levene test, significant differences in variance were identified in 13 courses (81.3%); in all of these cases, the standard deviation was greater in 2020 than in 2019 (Table 2, Fig. 1).

### Correlation of test scores between courses

**Fig. 2.**
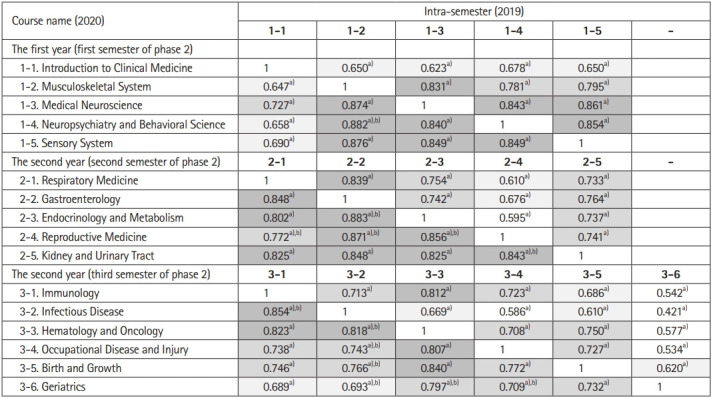
Intra-semester correlations of test scores. The values for 2020 are below the diagonal. The strength of the correlation was displayed using different shading (no shades: r <0.6, light gray: 0.6≤ r <0.7, medium gray: 0.7≤ r <0.8, dark gray: 0.8≤ r). ^a)^P<0.01. ^b)^Indicates a statistically significant increase between 2019 and 2020.

For both 2019 and 2020, most of the integrated, system-based curriculum test scores showed strong correlations, with coefficients of 0.6 or higher. In 2020, the correlation coefficient was generally even higher, with 30 (85.7%) pairs out of a total of 35 having a greater correlation coefficient in 2020, of which 13 (37.1%) showed a statistically significant increase (Fig. 2). Further, when analyzing the correlation between the first- and second-semester courses for second-year students, the correlation coefficient was greater in all 30 pairs (100.0%) in 2020 than in 2019. Of them, 18 (60.0%) showed a statistically significant increase (Table 3).

### Comparison between low-, middle-, and high-achieving students

After dividing students into low, middle, and high achievement groups based on overall major examination performance for a semester, we calculated the effect size of the difference between 2019 and 2020, while the sign (positive or negative) was maintained by not taking the absolute value. As a result, the average value of the effect sizes in all semesters was highest for low-achieving students, followed in descending order by middle-achieving and high-achieving students (Table 4). Specifically, low achievers showed positive values for the effect size for all 3 semesters, indicating a decline in test scores in 2020 compared to 2019.

## Discussion

### Key results

In this study, we compared students’ academic performance in integrated, system-based courses in the pre-clerkship curriculum before and after the COVID-19 pandemic to examine changes during the transition to full-scale online classes. In a majority of courses, the average test scores decreased, accompanied by an increase in variance compared to offline classes. The correlation of test scores between courses was mostly higher in 2020 in both intra- and inter-semester analyses. Finally, the decline in performance was most noticeable among low-achieving students compared to middle- or high-achieving students.

### Interpretation

Among the 16 courses in phase 2, the average test scores in 10 courses decreased significantly, and only 3 improved significantly. In general, our findings suggest that the transition to online classes due to COVID-19 has led to an overall decline in academic performance. Interestingly, in a meta-analysis prior to the COVID-19 pandemic, which included research published from 2000 to 2017, the knowledge outcomes of online learning were found to be at least equal or superior to those of offline learning in undergraduate medical education [8]. Therefore, when interpreting the findings of this study, both the pedagogical differences between online and offline formats in delivering content and overall changes in the broader educational environment caused by COVID-19 must be taken into account.

First, we must consider that the online transition of the formal curriculum due to COVID-19 was sudden, comprehensive, and compulsory. Faculty members were required to adapt to online teaching even if they were not provided with enough institutional support or were not skilled in technology, and students likewise had no choice but to study online. Further, because not all forms of teaching can be delivered online, hands-on practice (e.g., laboratory sessions or cadaver dissection) was inevitably reduced or discontinued. Additionally, social distancing greatly reduced opportunities for informal learning.

Nevertheless, it should be noted that the degree of decline in academic performance was not uniform across students. When the average of the effect sizes was calculated for each group (low-, middle-, and high-achieving students), with “average 2019 scores–average 2020 scores” used as a numerator, low-achieving students showed the highest positive value (i.e., the largest decline in test scores among the 3 groups). In contrast, high-achieving students showed the smallest positive value, or sometimes even a negative value (i.e., an increase in test scores). This stark difference between high- and low-achieving students could be attributed to the increased isolation caused by social distancing, which further amplified the importance of self-regulation in learning. In general, it is well known that struggling learners tend to show low self-regulation such as poor motivation and inefficient resource management. On the contrary, high-achieving students could have minimized the impact or even turned this crisis into an opportunity by utilizing a variety of motivational, cognitive, and metacognitive regulation strategies as well as appropriate resource management [9]. Moreover, since some interactive learning methods, such as team-based learning, are more beneficial to lower-achieving students than to high-achieving students in terms of knowledge acquisition [10], there is a possibility that the inevitable reduction or discontinuation of certain components in the formal curriculum may have been more damaging to already-struggling students.

### Comparison with previous studies

Considering that the integrated courses are relatively homogeneous in their content domains (i.e., clinical medicine) and assessment format (i.e., MCQs), it is common for multiple test scores to correlate with each other. The scores from the 16 courses consistently were highly correlated at the intra- and inter-semester levels. Moreover, unlike before the COVID-19 pandemic, these high correlations lasted until the second semester, after which students could spend about a month on vacation to review and revise their own learning strategies and behaviors. This suggests that students’ academic performance was “ossified” throughout this phase, which can be a critical problem, especially for lower-achieving students.

The negative consequences of this ossification can be viewed in 2 respects. One is an increase in the number of students who fail to progress. This is not only an unfavorable event for individual students, but also a managerial burden at the organizational level, given that remediation of struggling learners is resource-intensive work that requires significant time, performance, and expertise [11]. The other problem is that even if low achieving students progress to the clerkship phase, their weak academic foundation would pose future difficulties in learning advanced knowledge and skills. Theoretically, knowledge acquisition corresponds to the lowest level of Miller’s pyramid, “knows”, which forms the basis of subsequent higher-level performance such as “knows how”, “shows how”, and “does”. Empirically, on the medical education continuum, academic achievement in the previous phase has been identified as a major predictor of performance in the next phase, which McManus et al. [12] named the “academic backbone”. In the short term, provided that specific cognitive knowledge supports the basis of clinical reasoning as well as procedural skills, it is anticipated that students will continue to have difficulties in developing competence in the subsequent clerkship phase. Above all, in the long term, incompetent learners could pose a risk to patient safety.

### Limitations

First, in terms of research design, as we compared the academic achievement in online classes between 2019 and 2020, the results of the study may have been confounded by the characteristics of the cohorts of each year. Second, although data were collected across 16 courses and over a year and a half, this study was limited to a single institution. Third, while the COVID-19 pandemic continued throughout the 3 semesters of this study, its severity varied from one period to another. As a result, there were variations in institutional policies and student behavior, but not all of these micro-level changes were considered as variables in the analysis. Finally, 2020 was the first year of the transition to online classes due to the pandemic, and neither professors nor students were fully prepared. Therefore, our findings could be attributable to students’ level of adaptation and utilization of the online curriculum rather than online classes themselves.

### Suggestions

First, the overall decline in academic performance, which may occur when the curriculum is centered on recorded lectures, needs to be addressed. Improving the teaching quality—whether live or recorded—in delivering content would be the most basic and effective strategy, not only because the quality of recorded online lessons affects learners’ performance on examinations, but also because low-achieving students benefit the most from quality improvements [13]. Specifically, strategies for the effective use of videos, such as using interactive elements, managing cognitive overload, and considering technical requirements, should be considered.

Adding SDE components can be considered as a means of supplementing the impaired informal learning due to COVID-19. While asynchronous distance education is suitable for encouraging learners to cognitively participate in information processing, SDE is more advantageous for promoting psychological arousal and motivation [14]. As such, it would be most effective to use both in complementary ways. However, considering the high preference of students for recorded lectures, and the low probability of their active participation in live online lectures, it would not be appropriate to simply convert recorded lectures to a live format, even if they are delivered in a synchronous manner.

Second, the findings suggest the necessity of establishing a support system for the academic development of low-achieving students. The high correlation between tests implies that at-risk students can be predicted early with high probability. In particular, struggling in school has been linked to poor self-regulation in early medical students, and there is strong evidence for focusing remediation on assessing and improving self-regulation [11]. Therefore, preventive and proactive developmental approaches, focusing on developing students' personal and professional growth and lifelong learning skills, should be available in the early stages to prevent summative failure. More importantly, such an approach is more educationally desirable than a deficit-reactive approach, which has the risk of stigma or repeated failure even after remediation.

Third, future studies are necessary to clarify the causes and mechanisms of changes in academic performance in the online curriculum. For example, although most course components were largely the same except for the online transition between 2019 and 2020, differences in test conditions or examinee characteristics could have led to score improvements in some courses. Therefore, the use of experimental research design or statistical methods such as equating may be considered to draw more generalizable conclusions.

### Conclusion

This study identified a decline in students’ academic performance in the pre-clerkship curriculum that was transitioned to online classes because of COVID-19. Further studies from other institutions that have experienced similar changes and in-depth investigations into the causes of these phenomena should be conducted.

## Figures and Tables

**Table 1.** Characteristics of the courses

Course name	Year	Total lecture time (hr)	Total dissection time (hr)	No. of major exams
The first year (first semester of phase 2)				
Introduction to Clinical Medicine	2019	31	0	1
	2020	31	0	1
Musculoskeletal System	2019	59	60	3
	2020	63	60	2
Medical Neuroscience	2019	119	29	3
	2020	119	24	3
Neuropsychiatry and Behavioral Science	2019	53	0	2
	2020	55	0	2
Sensory System	2019	84	20	2
	2020	86	21	2
The second year (second semester of phase 2)				
Cardiovascular System	2019	73	8	2
	2020	79	6	2
Respiratory Medicine	2019	56	16	2
	2020	56	20	3
Gastroenterology	2019	110	22	3
	2020	104	20	3
Endocrinology and Metabolism	2019	47	6	2
	2020	47	6	2
Reproductive Medicine	2019	51	24	1
	2020	52	24	2
Kidney and Urinary Tract	2019	58	15	3
	2020	58	19	2
The second year (third semester of phase 2)				
Immunology	2019	31	0	1
	2020	33	0	1
Infectious Disease	2019	61	16	1
	2020	61	7	1
Hematology and Oncology	2019	63	9	2
	2020	63	11	2
Occupational Disease and Injury	2019	22	4	1
	2020	22	3	1
Birth and Growth	2019	92	2	2
	2020	90	6	2
Geriatrics	2019	22	0	1
	2020	24	0	1

**Table 2.** Differences between 2019 and 2020 test scores

Course name	Mean±SD		Levene test		t-test		Cohen D
	2019	2020	F	P-value	t-value	P-value	
The first year (first semester of phase 2)							
Introduction to Clinical Medicine	87.73±5.17	82.93±8.08	21.888	<0.001	5.038	<0.001	0.71
Musculoskeletal System	79.95±8.00	78.04±10.77	0.028	0.028	1.433	0.154	0.20
Medical Neuroscience	78.39±8.63	71.84±11.95	7.530	0.007	4.464	<0.001	0.63
Neuropsychiatry and Behavioral Science	80.21±7.23	79.59±7.40	0.225	0.636	0.599	0.55	0.08
Sensory System	85.05±7.29	79.00±10.12	9.820	0.002	4.873	<0.001	0.69
The second year (second semester of phase 2)							
Cardiovascular System	75.43±7.34	72.04±9.13	9.178	0.003	3.084	<0.001	0.41
Respiratory Medicine	83.44±6.67	88.78±6.55	0.000	0.994	-6.041	<0.001	-0.81
Gastroenterology	84.88±5.90	79.55±8.17	15.826	<0.001	5.539	<0.001	0.75
Endocrinology and Metabolism	81.31±6.96	85.20±8.62	5.402	0.021	-3.695	<0.001	-0.50
Reproductive Medicine	76.15±8.28	84.65±8.84	0.956	0.329	-7.443	<0.001	-0.99
Kidney and Urinary Tract	84.82±6.16	78.39±8.40	7.781	0.006	6.461	<0.001	0.87
The second year (third semester of phase 2)							
Immunology	85.05±8.11	82.21±10.72	9.526	0.002	2.218	0.028	0.30
Infectious Disease	77.49±8.11	79.29±10.23	4.518	0.035	-1.448	0.149	-0.20
Hematology and Oncology	85.51±5.40	83.00±7.01	7.999	0.005	3.329	0.001	0.45
Occupational Disease and Injury	75.66±6.97	71.69±9.97	13.861	<0.001	3.418	0.001	0.46
Birth and Growth	88.84±5.17	84.64±6.41	4.711	0.031	5.348	<0.001	0.72
Geriatrics	90.96±4.77	86.84±8.16	26.898	<0.001	4.550	<0.001	0.62

SD, standard deviation.

**Table 3.** Inter-semester correlations of test scores

Courses in the first semester of the second year	Year	Courses in the second semester of the second year					
		Immunology	Infectious Disease	Hematology and Oncology	Occupational Disease and Injury	Birth and Growth	Geriatrics
Respiratory Medicine	2019	0.645^a)^	0.543^a)^	0.692^a)^	0.639^a)^	0.724^a)^	0.506^a)^
	2020	0.767^a)^	0.777^a),b)^	0.756^a)^	0.682^a)^	0.739^a)^	0.686^a)^
Gastroenterology	2019	0.565^a)^	0.410^a)^	0.621^a)^	0.641^a)^	0.755^a)^	0.528^a)^
	2020	0.792^a),b)^	0.836^a),b)^	0.843^a),b)^	0.782^a),b)^	0.824^a)^	0.698^a)^
Endocrinology and Metabolism	2019	0.578^a)^	0.456^a)^	0.608^a)^	0.621^a)^	0.666^a)^	0.504^a)^
	2020	0.797^a),b)^	0.820^a),b)^	0.822^a),b)^	0.726^a)^	0.803^a),b)^	0.692^a)^
Reproductive Medicine	2019	0.701^a)^	0.562^a)^	0.666^a)^	0.623^a)^	0.701^a)^	0.517^a)^
	2020	0.789^a)^	0.769^a),b)^	0.800^a),b)^	0.717^a)^	0.769^a)^	0.644^a)^
Kidney and Urinary Tract	2019	0.580^a)^	0.423^a)^	0.613^a)^	0.533^a)^	0.715^a)^	0.600^a)^
	2020	0.833^a),b)^	0.844^a),b)^	0.854^a),b)^	0.769^a),b)^	0.817^a)^	0.744^a)^

a) P<0.01.

b) Indicates a statistically significant increase between 2019 and 2020.

**Table 4.** Effect sizes for students with low, middle, and high academic achievement

Variable	Cohen D			
	All students	Low-achieving students	Middle-achieving students	High-achieving students
The first year (first semester of phase 2)				
Introduction to Clinical Medicine	0.71^a)^	1.56^a)^	1.20^a)^	0.55^b)^
Musculoskeletal System	0.20	0.59^b)^	0.38	0.15
Medical Neuroscience	0.63^a)^	1.26^a)^	1.20^a)^	1.37^a)^
Neuropsychiatry and Behavioral Science	0.08	0.10	0.15	0.57^b)^
Sensory System	0.69^a)^	1.38^a)^	1.41^a)^	1.33^a)^
Mean		0.98	0.87	0.80
The second year (second semester of phase 2)				
Respiratory Medicine	-0.81^a)^	-1.17^a)^	-1.64^a)^	-2.21^a)^
Gastroenterology	0.75^a)^	1.65^a)^	1.77^a)^	0.71^a)^
Endocrinology and Metabolism	-0.50^a)^	-0.25	-1.59^a)^	-2.32^a)^
Reproductive Medicine	-0.99^a)^	-1.02^a)^	-1.63^a)^	-2.57^a)^
Kidney and Urinary Tract	0.87^a)^	1.59^a)^	1.60^a)^	1.21^a)^
Mean		0.16	-0.30	-1.04
The second year (third semester of phase 2)				
Immunology	0.30^b)^	0.60^a)^	0.12	-0.13
Infectious Disease	-0.20	0.33	-0.87^a)^	-1.10^a)^
Hematology and Oncology	0.45^a)^	0.84^a)^	1.24^a)^	-0.05
Occupational Disease and Injury	0.46^a)^	1.07^a)^	0.47	0.13
Birth and Growth	0.72^a)^	1.19^a)^	1.69^a)^	0.87^a)^
Geriatrics	0.62^a)^	1.17^a)^	0.84^a)^	0.02
Mean		0.87	0.58	-0.04

a) P<0.01.

b) P<0.05.

## Footnotes

**Data availability**
Data files are available from Harvard Dataverse: https://doi.org/10.7910/DVN/OBZCIT Dataset 1. Students’ performance records of 16 courses in 2019 and 2020.

## Supplementary materials

Supplement 1. Audio recording of the abstract.
